# CACUL1 reciprocally regulates SIRT1 and LSD1 to repress PPARγ and inhibit adipogenesis

**DOI:** 10.1038/s41419-017-0070-z

**Published:** 2017-12-11

**Authors:** Min Jun Jang, Ui-Hyun Park, Jeong Woo Kim, Hanbyeul Choi, Soo-Jong Um, Eun-Joo Kim

**Affiliations:** 1 Department of Molecular Biology, Dankook University, Cheonan-si, Chungnam 31116 Korea; 2 Department of Integrative Bioscience and Biotechnology, Sejong University, 209 Neungdong-ro, Gwangjin-gu, Seoul 05006 Korea

## Abstract

Peroxisome proliferator-activated receptor γ (PPARγ) is the master regulator of adipocyte differentiation and is closely linked to the development of obesity. Despite great progress in elucidating the transcriptional network of PPARγ, epigenetic regulation of this pathway by histone modification remains elusive. Here, we found that CDK2-associated cullin 1 (CACUL1), identified as a novel SIRT1 interacting protein, directly bound to PPARγ through the co-repressor nuclear receptor (CoRNR) box 2 and repressed the transcriptional activity and adipogenic potential of PPARγ. Upon CACUL1 depletion, less SIRT1 and more LSD1 were recruited to the PPARγ-responsive gene promoter, leading to increased histone H3K9 acetylation, decreased H3K9 methylation, and PPARγ activation during adipogenesis in 3T3-L1 cells. These findings were reversed upon fasting or resveratrol treatment. Further, gene expression profiling using RNA sequencing supported the repressive role of CACUL1 in PPARγ activation and fat accumulation. Finally, we confirmed CACUL1 function in human adipose-derived stem cells. Overall, our data suggest that CACUL1 tightly regulates PPARγ signaling through the mutual opposition between SIRT1 and LSD1, providing insight into its potential use for anti-obesity treatment.

## Introduction

The worldwide increase in obesity is significantly correlated with various metabolic diseases, such as type II diabetes, hypertension, cardiovascular disease, and cancer^1^. Obesity is closely associated with increased adipocyte number and size, and excessive lipid accumulation in adipose tissues due to an imbalance between energy intake and expenditure^2,3^. Therefore, any strategies for inhibiting adipocyte differentiation (known as adipogenesis) that contribute to a reduction in adipose tissue attract considerable interest as a potential treatment for obesity. Adipogenesis is the multistep process of growth arrest, clonal expansion, and terminal differentiation of preadipocytes into mature adipocytes, accompanied by genetic changes in lipid synthesis and storage^4^. The adipogenic process is tightly controlled by an elaborate network of transcription factors, of which peroxisome proliferator-activated receptor γ (PPARγ) is the central regulator of adipogenesis^5–7^.

PPARγ is a ligand-activated transcription factor that belongs to the nuclear receptor (NR) superfamily^8^. When PPARγ is bound by a ligand, such as endogenous arachidonic acid metabolites or the synthetic agonist thiazolidinedione (TZD), it forms a heterodimer with the retinoid X receptor (RXR), which allows binding to the PPAR-response element (PPRE) in the promoter of adipogenic genes, such as adipocyte protein 2 (*aP2*, also called fatty acid binding protein 4 or *Fabp4*) and lipoprotein lipase (*LPL*)^9^. The transcriptional activity of PPARγ is diversely regulated through associations with various coactivators and corepressors, many of which are histone-modifying enzymes that target histone acetylation or methylation^10^.

SIRT1, a member of the sirtuin family of proteins, is a NAD^+^-dependent class III histone deacetylase that has been implicated in playing a pivotal role in maintaining metabolic homeostasis and regulating diverse physiology in mammals^11,12^. SIRT1 attenuates adipogenesis and promotes fat mobilization by repressing PPARγ^13^, whereas adipose-specific deficiency of SIRT1 in mice enhances metabolic dysfunction and insulin resistance^14^. Furthermore, SIRT1 knockdown in adipose tissue leads to histone hyperacetylation and induces inflammation and macrophage influx into white adipose tissue^15,16^. Despite the potential importance of SIRT1 in adipogenesis and obesity, the epigenetic mechanism underlying PPARγ repression is largely unknown. Recently, lysine-specific histone demethylase 1 (LSD1) has been reported to be involved in modulating adipogenesis in 3T3-L1 cells by removing methyl groups from dimethylated H3 lysine 9 (H3K9me2), a repressive histone mark^17^. Previously, we showed that CDK2-associated cullin 1 (CACUL1) associates with LSD1 to suppress the LSD1-enhanced transcriptional activities of estrogen receptor alpha (ERα) and androgen receptor (AR)^18,19^. Therefore, addressing the cross-talk mechanisms with other histone-modifying enzymes, such as LSD1, is of great interest.

In this study, we report that CACUL1, identified as a novel SIRT1 interacting protein, physically interacts with PPARγ and represses its transcriptional activity, suppressing adipocyte differentiation. Further epigenetic studies indicated that CACUL1 functionally associates with SIRT1 and LSD1 at the PPARγ-responsive gene promoter, and regulates the level of histone H3K9 acetylation and methylation to repress PPARγ during adipocyte differentiation. This role for CACUL1 was confirmed by RNA sequencing (RNA-seq) analysis and further demonstrated in human adipose-derived stem cells (ADSCs).

## Results

### CACUL1 is identified as a SIRT1 binding protein

**Fig. 1:**
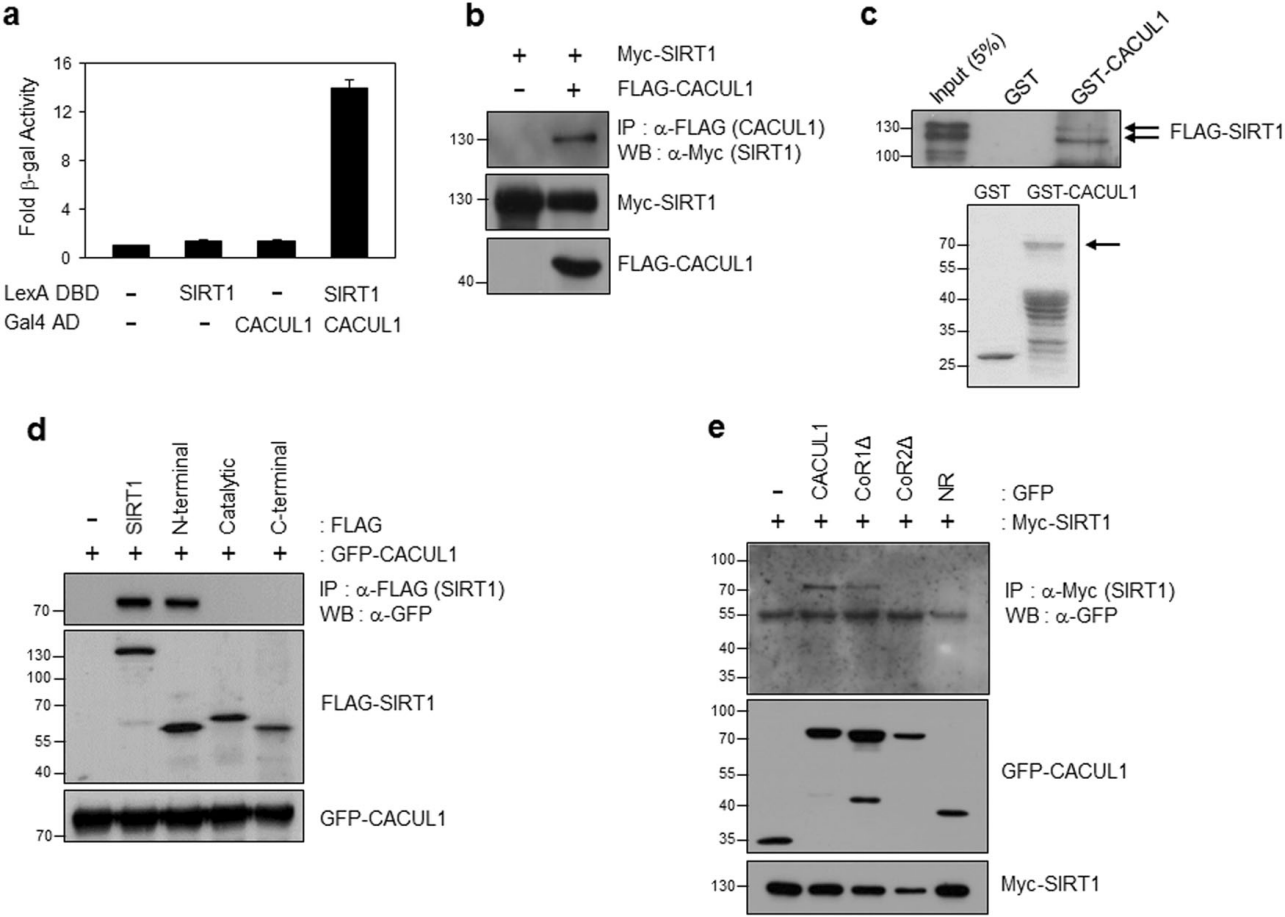
**CACUL1 is identified as a SIRT1-binding protein** **a** Yeast two-hybrid assay. L40 cells were transformed with LexA DNA binding domain (DBD)-fused SIRT1 (in pBTM116 vector) and Gal4 activating domain (AD)-fused CACUL1. The interaction was measured by β-galactosidase (β-gal) assays. The data were processed in accordance with statistical analysis methods as means±standard deviation (SD) of triplicate experiments. **b** Immunoprecipitation (IP) analysis. HEK293 cells were co-transfected with Myc-SIRT1 and FLAG-CACUL1 or FLAG vector. IP using an anti-FLAG antibody was followed by western blotting (WB) with an anti-Myc antibody. **c** Glutathione S-transferase (GST) pull-down assay. GST or GST-CACUL1 was incubated with in vitro translated FLAG-SIRT1. The interaction was monitored by WB using an anti-FLAG antibody. **d**, **e** Mapping of domains responsible for the interaction. HEK293 cells were transfected with green fluorescent protein (GFP)-CACUL1 and FLAG-tagged SIRT1 deletions, or Myc-SIRT1 and GFP-fused CACUL1 fragments. SIRT1 mapping was determined by IP using an anti-FLAG antibody and WB using an anti-GFP antibody (**d**). CACUL1 mapping was done by IP using an anti-Myc antibody and WB using an anti-GFP antibody (**e**)

From yeast two-hybrid screening using a human HeLa cDNA library and SIRT1 as the bait, we identified CACUL1 (also called CAC1)^20^, as a putative SIRT1 interacting protein (Fig. 1a). We previously showed that CACUL1 interacts with NRs including ERα, AR, and retinoic acid receptor alpha, and inhibits their transcriptional activities^18,19,21^. However, the physiological function of CACUL1 remains largely unknown. To validate the physical interaction of CACUL1 with SIRT1, FLAG-CACUL1 and Myc-SIRT1 were transfected into HEK293 cells. Subsequent immunoprecipitation (IP) using an anti-FLAG antibody followed by western blotting (WB) with an anti-Myc antibody demonstrated the interaction in vivo (Fig. 1b). This interaction was confirmed by reverse co-IP (Supplementary Figure S1A). Further glutathione S-transferase (GST) pull-down assays indicated the direct binding of CACUL1 to SIRT1 in vitro (Fig. 1c). To map the SIRT1 region responsible for CACUL1 binding, we coexpressed the FLAG-tagged N-terminal, C-terminal, and catalytic domain of SIRT1 deletions (Supplementary Figure S1B) with GFP-tagged CACUL1 into HEK293 cells. As shown by IP/WB, the N-terminal region of SIRT1 was responsible for the interaction (Fig. 1d).

Our previous study revealed that CACUL1 harbors two conservative co-repressor NR (CoRNR) boxes, of which box 2 is critical for the interaction and repression of NRs (Supplementary Figure S1C). Based on this finding, we attempted to map the CACUL1 region required for SIRT1 interaction by coexpressing green fluorescent protein (GFP)-CACUL1 lacking CoRNR box 1 (CoR1∆), CoRNR box 2 (CoR2∆), or NR box mutants, together with Myc-SIRT1, into HEK293 cells. Subsequent IP/WB revealed no SIRT1 interactions with CACUL1 mutants lacking CoRNR box 2 or the NR fragment (Fig. 1e), suggesting that the CoRNR box 2 of CACUL1 is critical for its interaction with SIRT1. The result was further confirmed using a substitution mutant (AxxTT: amino acids LQSIV were changed to AQSTT in the CoRNR box 2) (Supplementary Figure S1D).

### CACUL1 interacts with PPARγ

**Fig. 2:**
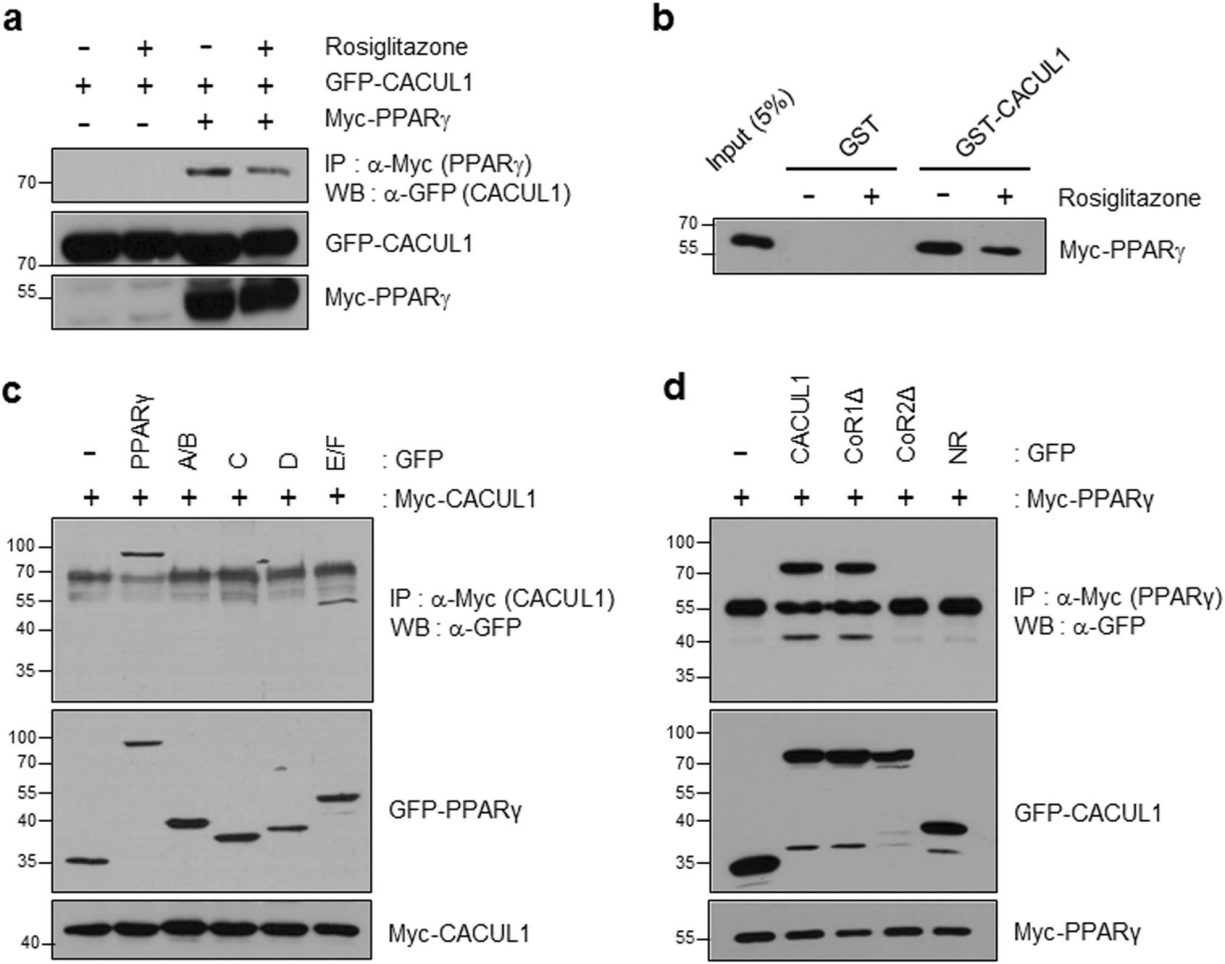
**CACUL1 interacts with PPARγ** **a** IP analysis. HEK293 cells were transfected as indicated. IP using an anti-Myc antibody was followed by WB using an anti-GFP antibody. **b** GST pull-down assay. GST or GST-CACUL1 was incubated with in vitro translated Myc-PPARγ in the presence of the PPARγ agonist rosiglitazone. **c**, **d** Mapping of domains of PPARγ (**c**) and CACUL1 (**d**) responsible for the interaction. HEK293 cells were transfected as indicated. IP/WB was performed as described

Given our previous study and mapping data, we addressed whether CACUL1 participates in SIRT1-mediated NR regulation. For this purpose, we chose PPARγ, another NR superfamily member, because it is known to be repressed by SIRT1^13^. As shown by IP/WB analysis using GFP-CACUL1 and Myc-PPARγ, we observed the interaction, which was slightly diminished in the presence of the ligand rosiglitazone (Fig. 2a). This interaction was confirmed in vitro by GST pull-down assay (Fig. 2b). In addition, we explored mapping the domain of PPARγ required for CACUL1 binding. We coexpressed Myc-tagged CACUL1 with GFP-fused PPARγ deletion mutants into HEK293 cells. Subsequent co-IP showed that the PPARγ-E/F domain was responsible for CACUL1 association (Fig. 2c). Similar to its interaction with SIRT1, CACUL1 interacted with PPARγ through CoRNR box 2 (Fig. 2d), suggesting that CACUL1 may contribute to SIRT1-mediated PPARγ repression. Co-IP using a substitution mutant (AxxTT) supported the essential role of the CoRNR box 2 for the interaction of CACUL1 with PPARγ (Supplementary Figure S2).

### CACUL1 represses the transcriptional activity of PPARγ and adipocyte differentiation

**Fig. 3:**
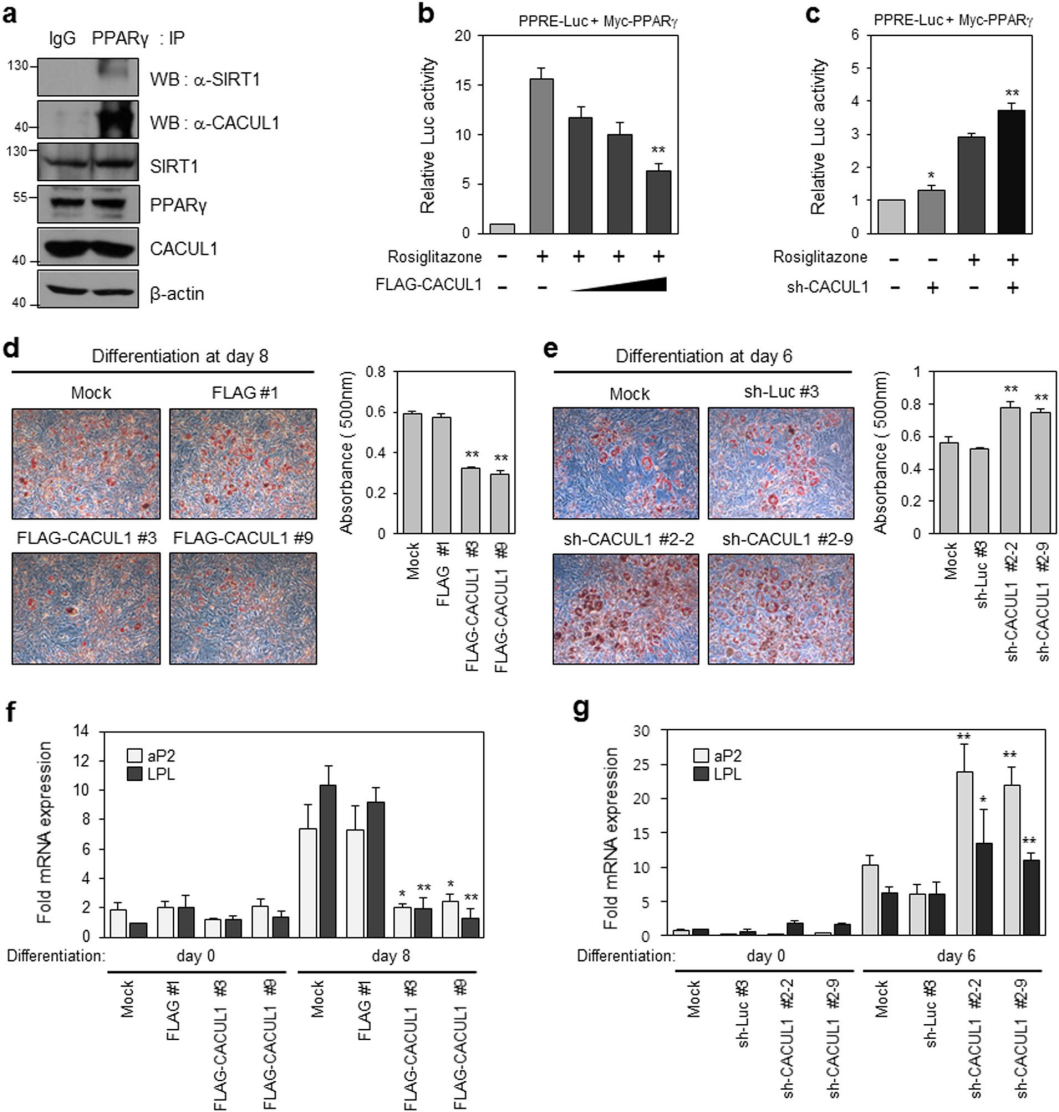
**CACUL1 represses PPARγ-mediated transcriptional activity and adipogenesis** **a** Endogenous interactions among PPARγ, SIRT1, and CACUL1 in 3T3-L1 preadipocytes. IP was performed using an anti-PPARγ antibody and bound protein complex was visualized with WB using an anti-SIRT1 or anti-CACUL1 antibody. **b** Effect of CACUL1 overexpression on PPARγ transcriptional activity. HEK293 cells were transiently transfected with a PPRE-Luc reporter and increasing amounts of FLAG-CACUL1 with Myc-PPARγ in the presence (or absence) of 5 µm rosiglitazone. Cell extracts were subjected to luciferase assays. Data represent means±standard deviation (SD; *n* = 3) from three independent experiments (**P < 0.01). **c** Effect of CACUL1 depletion on PPARγ activity. Luciferase assays were performed as described using extracts of HEK293 transfected with sh-CACUL1. Data represent means±SD from three independent experiments (*P < 0.05, **P < 0.01). **d** Effect of CACUL1 overexpression on adipogenesis. 3T3-L1 cells were transfected with FLAG-CACUL1 (or FLAG) and selected against G418 for stable expression of CACUL1. Selected cells were subjected to differentiation for 8 days. Lipid accumulation was visualized by Oil Red O staining and light microscopy at ×100. The amount of lipid was quantified using a spectrophotometer at 500 nm. Data represent means±SD from three independent experiments (**P < 0.01). **e** Effect of CACUL1 knockdown on adipogenesis. 3T3-L1 cells were transfected with sh-CACUL1 (or sh-Luciferase) and selected against Hygromycin B. (**P < 0.01). **f**, **g** Effects of CACUL1 on the expression of PPARγ target genes. Stable 3T3-L1 cells with either CACUL1 overexpression (**f**) or knockdown (**g**) were subjected to differentiation for 6−8 days. Subsequent RT-qPCR was performed using primer sets for two PPARγ target genes associated with adipogenesis, *aP2* (also called *Fabp4*) and *LPL*. Fold mRNA expression was normalized to GAPDH expression (*P < 0.05, **P < 0.01)

To evaluate the physiological significance of the physical interaction observed above, we investigated whether CACUL1 regulates PPARγ-mediated transcriptional activity. Prior to examining the effects of CACUL1, we confirmed the endogenous interaction of these proteins in preadipocyte 3T3-L1 cells. Indeed, both SIRT1 and CACUL1 were detected by IP with anti-PPARγ antibody and WB using each antibody, supporting the endogenous PPARγ interaction with SIRT1 and CACUL1 (Fig. 3a). For transcription assays, HEK293 cells were cotransfected with a PPRE-luciferase reporter and PPARγ, and increasing amounts of CACUL1 in the presence of rosiglitazone. As shown in Fig. 3b, CACUL1 strongly repressed rosiglitazone-induced PPARγ transcriptional activity in a concentration-dependent manner. Supporting this result, augmented transcriptional activity of PPARγ was observed upon CACUL1 depletion (Fig. 3c). The CACUL1-mediated PPARγ regulation prompted us to address whether CACUL1 is functionally linked to PPARγ-activated adipocyte differentiation. For this purpose, we first generated 3T3-L1 cells stably expressing FLAG-CACUL1 (Supplementary Figure S3A) or sh-CACUL1 (Supplementary Figure S3B), for which expression was monitored by WB using an anti-FLAG or anti-CACUL1 antibody, respectively. Adipogenesis was measured by Oil Red O staining 6–8 days after treatment with a differentiation-inducing mixture containing rosiglitazone. As shown in Fig. 3d and e, fat accumulation was significantly downregulated in the FLAG-CACUL1 stable cells and upregulated in CACUL1-depleted cells. Consistently, CACUL1 overexpression led to a remarkable reduction in the expression of the PPARγ target genes, *aP2* and *LPL*, as determined by reverse transcription and quantitative PCR (RT-qPCR) (Fig. 3f). This effect was abolished in CACUL1 knockdown cells (Fig. 3g). Collectively, our observations suggest that CACUL1 inhibits PPARγ-mediated adipogenesis in 3T3-L1 cells by repressing the transcriptional activity of PPARγ.

### CoRNR Box 2 of CACUL1 is required for PPARγ inactivation

**Fig. 4:**
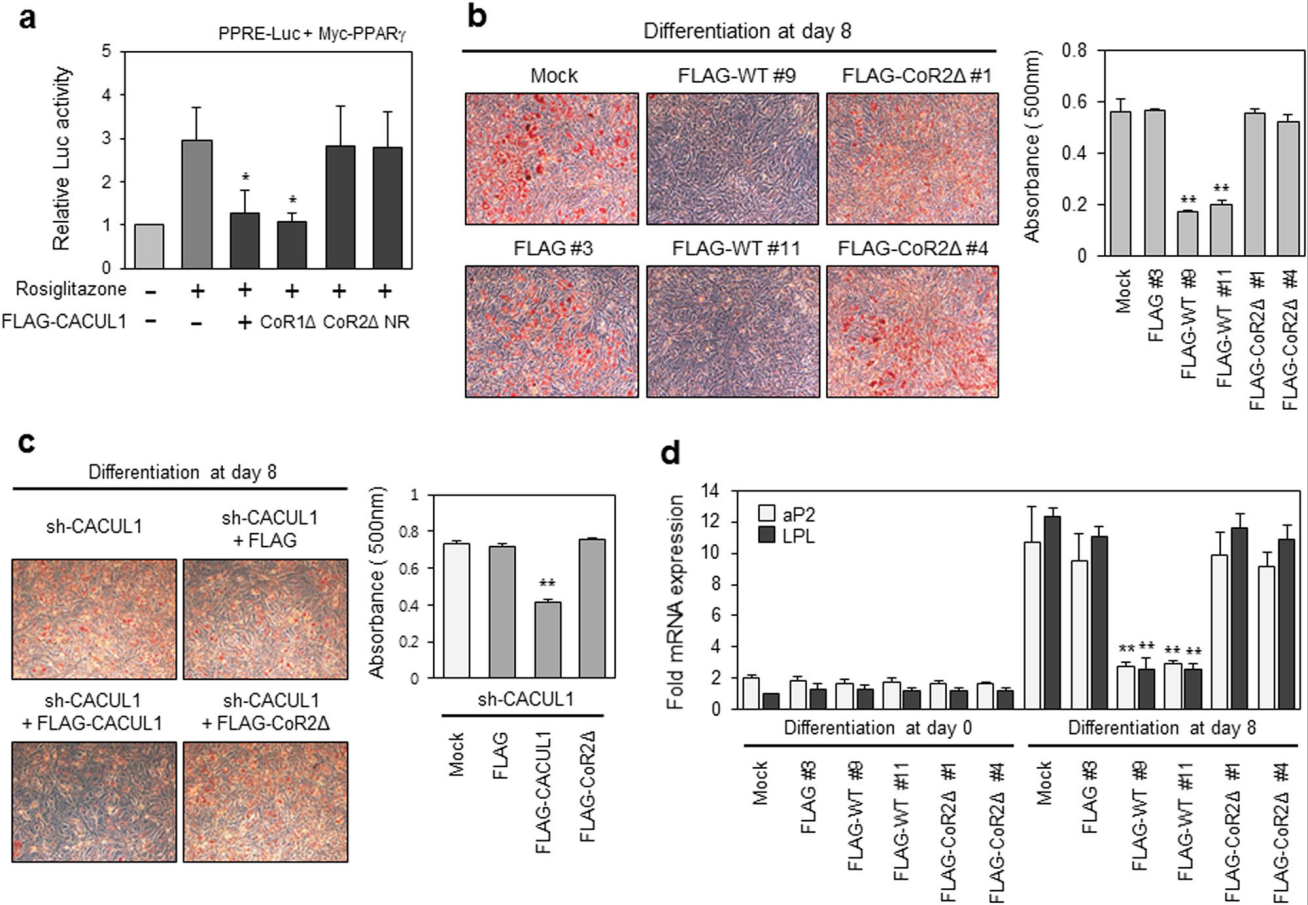
**CoRNR box 2 is required for the repressive function of CACUL1** **a** Effect of the CoRNR box 2 motif on CACUL1-mediated repression. HEK293 cells were transfected with a PPRE-Luc reporter, Myc-PPARγ, and FLAG-CACUL1 (or its mutants) in the presence of 5 µm rosiglitazone. Data represent means±SD from three independent experiments (*P < 0.05). **b** Effect on adipogenesis. Stable 3T3-L1 cells were selected as described, subjected to differentiation for 8 days, Oil red O staining, and extraction for measuring lipid amounts (**P < 0.01). **c** Effect of CoRNR box 2 motif under CACUL1 knockdown conditions. Stable 3T3-L1 cells with depleted CACUL1 were secondarily selected with FLAG-CACUL1 or FLAG-CoR2Δ (**P < 0.01). **d** Effect on the expression of PPARγ target genes. Stable 3T3-L1 cells as indicated were subjected to mRNA extraction for RT-qPCR. Data indicate means±SD from three independent experiments (**P < 0.01)

To confirm that the CACUL1 interaction with SIRT1 and PPARγ is critical for suppressing PPARγ activity, we used a CACUL1 mutant lacking CoRNR box 2 (CoR2Δ), which is defective for the interaction. Luciferase reporter assays demonstrated that wild-type (WT) CACUL1 and mutants lacking CoRNR box 1 (CoR1∆) strongly inhibited the rosiglitazone-induced transcriptional activity of PPARγ, whereas CoR2∆ and NR mutants failed to suppress PPARγ activity (Fig. 4a). In addition, luciferase assays using a substitution mutant (AxxTT) indicated that interaction via the CoRNR box2 is critical for PPARγ repression (Supplementary Figure S4A). To further explore the effects of CoRNR box 2 on PPARγ activation, we generated stable 3T3-L1 cells expressing FLAG-CoR2∆, and observed its expression by WB using an anti-FLAG antibody (Supplementary Figure S4B). Oil red O staining indicated that CoRNR box 2 is required for blocking PPARγ-mediated adipogenesis (Fig. 4b). Additional rescue experiments were performed to confirm the requirement of CoRNR box 2 for CACUL1 function. Stable CACUL1-depleted 3T3-L1 cells were secondly selected with FLAG-WT or a FLAG-CoR2∆ mutant (Supplementary Figure S4C), and subjected to differentiation for 8 days. Strong lipid accumulation under the CACUL1 knockdown condition was greatly inhibited by introducing WT CACUL1, but not by introducing mutant CoR2∆ (Fig. 4c). Consistently, the CACUL1 mutant lacking CoRNR box 2 was unable to suppress the expression of PPARγ target genes on Differentiation Day 8 (Fig. 4d). Overall, our data suggest that the CoRNR box 2 of CACUL1, responsible for CACUL1 binding to SIRT1 and PPARγ, is critical for repressing the transcriptional activity and adipogenic potential of PPARγ.

### CACUL1 reciprocally regulates SIRT1 and LSD1 for epigenetic repression of PPARγ

**Fig. 5:**
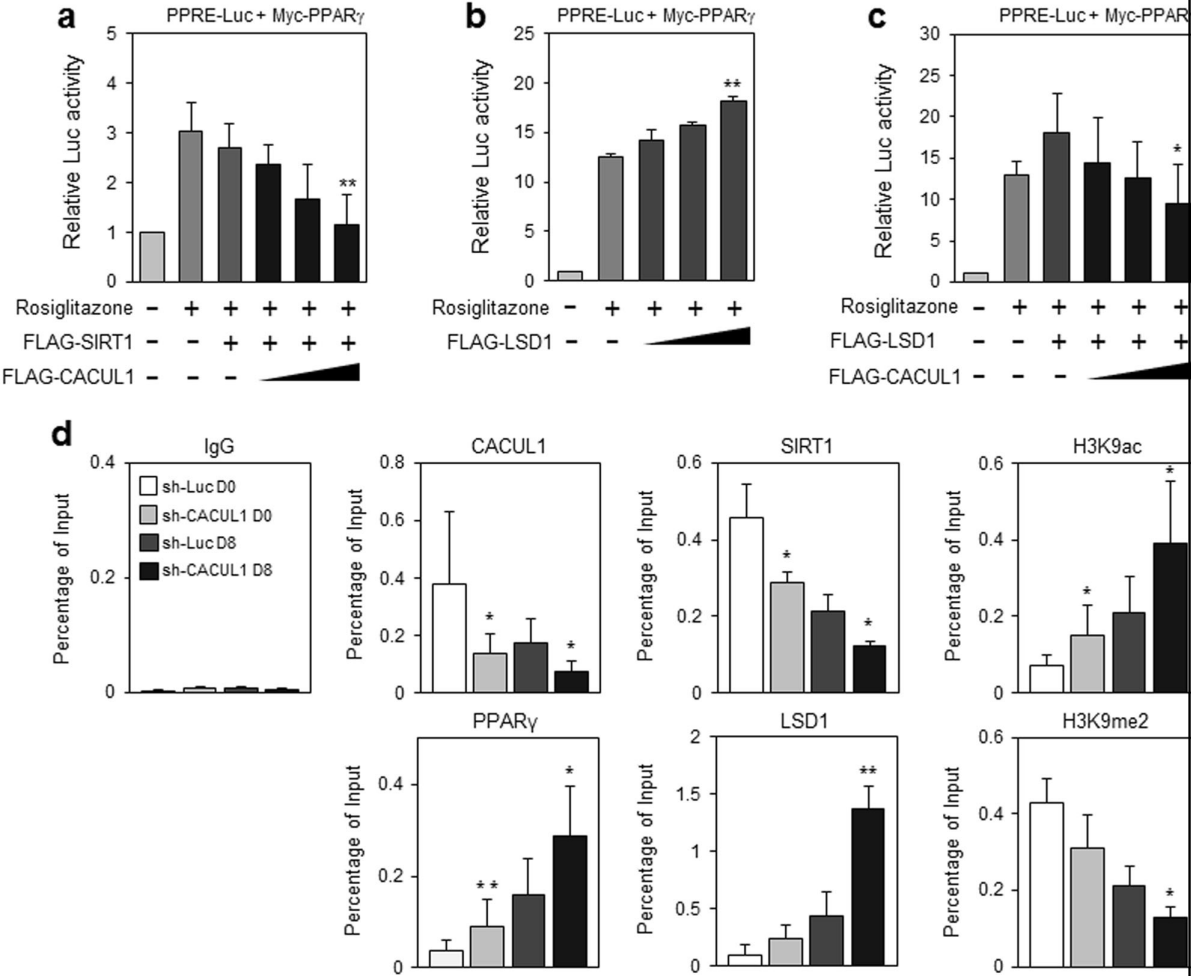

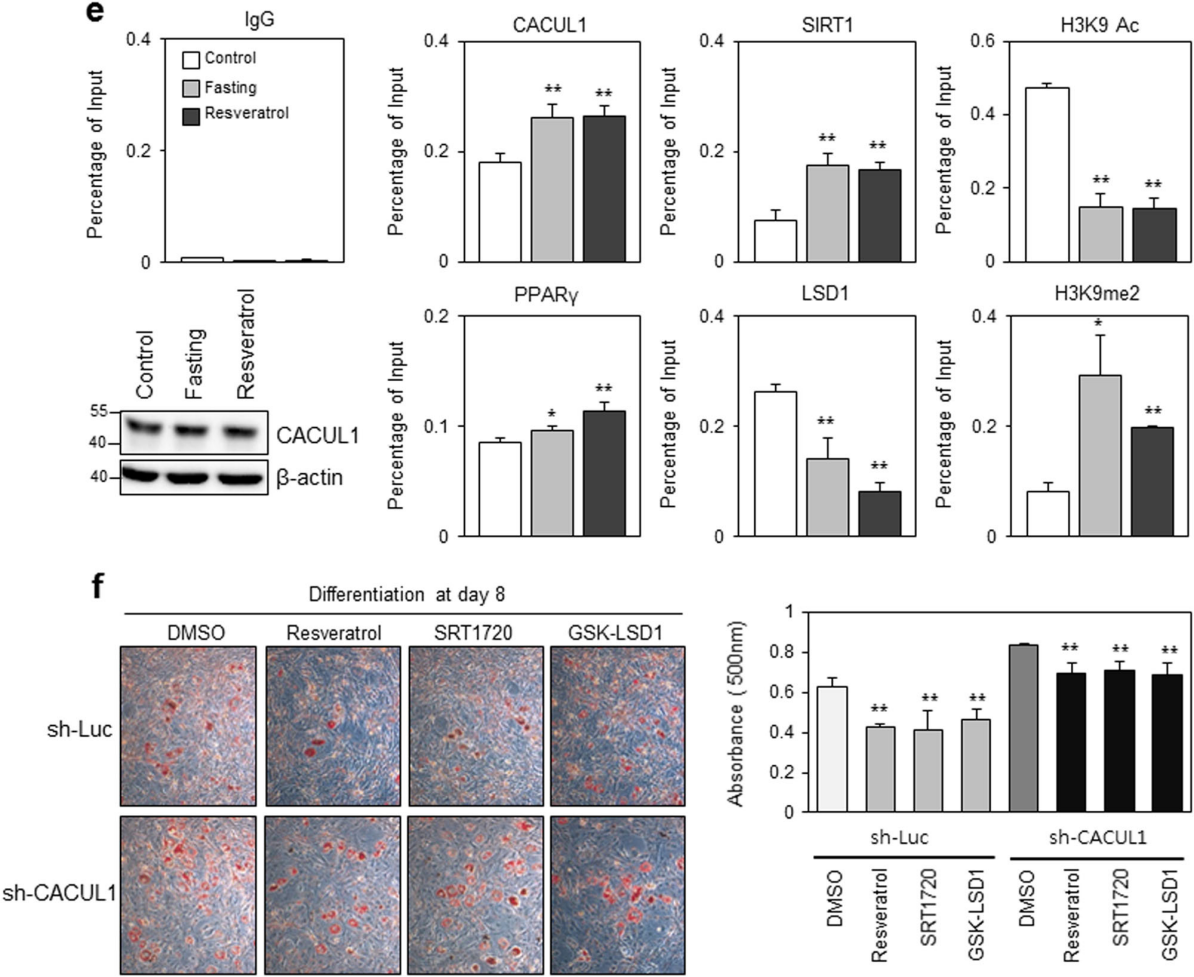
**CACUL1 reciprocally regulates SIRT1 and LSD1 for epigenetic repression of PPARγ** **a** Cooperation of CACUL1 and SIRT1 to mediate PPARγ repression. HEK293 cells were transfected as indicated and were subjected to extraction for luciferase assays. Data indicate means±SD from three independent experiments (**P < 0.01). **b** Effect of LSD1 on PPARγ activation. Transfections and luciferase assays were performed as indicated (**P < 0.01). **c** Negative effect of CACUL1 on LSD1-enhanced PPARγ activity. (*P < 0.05). **d** Effect of CACUL1 depletion on H3K9 modifications during adipogenesis. Stable 3T3-L1 cells depleted of CACUL1 were subjected to differentiation for 8 days and chromatin IP (ChIP) assays using a primer set specific for the distal PPARγ-response site within the *aP2* promoter and the indicated antibodies (H3K9ac and H3K9me, acetylation and demethylation at lysine 9 of histone H3, respectively). Data indicate means±SD from three independent experiments (*P < 0.05) (**P < 0.01). **e** Effect of fasting and resveratrol. After culturing undifferentiated 3T3-L1 cells in low-glucose DMEM (5.5 mm glucose; fasting condition) or treating with resveratrol (25 μm) for 24 h, ChIP assays were performed using the indicated antibodies. Data indicate means±SD from three independent experiments (*P < 0.05, **P < 0.01). CACUL1 expression is displayed by WB under these conditions. **f** Effect of resveratrol, SRT1720, or GSK-LSD1 treatment on adipogenesis under CACUL1-depleted conditions. CACUL1-depleted 3T3-L1 stable cells were treated with SIRT1 activators (resveratrol, 25 μm; SRT1720, 2.5 μm) or an LSD1 inhibitor (GSK-LSD1, 25 μm) and subjected to differentiation for 8 days. Lipid accumulation was visualized and measured as described (**P < 0.01)

Luciferase reporter assays using PPRE-Luc indicated that CACUL1 cooperated with histone deacetylase SIRT1 to repress PPARγ (Fig. 5a). In a previous study, we reported that CACUL1 associates with LSD1 to suppress the LSD1-mediated transcriptional activities of ERα and AR^18,19^. We further determined whether histone demethylase LSD1 plays a role in this complex regulation. Luciferase assays revealed that LSD1 enhanced the transcriptional activity of PPARγ (Fig. 5b). LSD1-enhanced PPARγ activation was significantly impaired by CACUL1 overexpression (Fig. 5c) and stimulated by CACUL1 depletion (Supplementary Figure S5A), suggesting a negative role for CACUL1 in LSD1-mediated PPARγ activation.

Positive or negative cooperation of CACUL1 with SIRT1 or LSD1 was determined by monitoring histone modifications at PPARγ target gene promoters. For this purpose, 3T3-L1 cells stably expressing sh-CACUL1 were subjected to chromatin immunoprecipitation (ChIP)-qPCR analysis using a primer set specific for a distal PPAR-responsive site in the *aP2* promoter and the indicated antibodies. Upon CACUL1 depletion during adipogenesis, SIRT1 recruitment to the promoter decreased, leading to a greater accumulation of histone H3K9 acetylation (H3K9ac). In contrast, the recruitment of PPARγ and LSD1 greatly increased, accompanied by a large reduction in dimethylated histone H3K9 (H3K9me2) in CACUL1-depleted cells (Fig. 5d). These data were supported by additional ChIP-qPCR assays targeting a proximal PPAR-responsive site in the *aP2* promoter (Supplementary Figure S5B). The site specificity was confirmed by targeting a non-PPAR target site as the negative control (Supplementary Figure S5C). Under SIRT1-activating conditions, which were achieved by fasting or resveratrol treatment without affecting CACUL1 expression, CACUL1 and SIRT1 recruitment was significantly enhanced and the H3K9ac level was greatly impaired, whereas reduced LSD1 binding to the *aP2* promoter was followed by increased H3K9me2 levels in 3T3-L1 cells (Fig. 5e). Overall, these data suggest that CACUL1 oppositely regulates two histone-modifying enzymes, SIRT1 and LSD1, leading to reduced H3K9 acetylation (active histone code) and increased H3K9 methylation (repressive histone code), resulting in the epigenetic suppression of PPARγ activity.

The reciprocal roles of SIRT1 and LSD1 in the CACUL1-mediated suppression of adipogenesis were further demonstrated by treating sh-Luc control or CACUL-depleted 3T3-L1 cells with SIRT1 activators (resveratrol, SRT1720) or an LSD1 inhibitor (GSK-LSD1). Subsequent Oil Red O staining demonstrated that these treatments reduced fat storage in control cells, but the effect was significantly reduced in CACUL1-depleted cells: from 32.4% repression in control cells to 17.2% in CACUL1-depleted cells with resveratrol; from 34.2 to 15.2% with SRT1720; and from 26.2 to 17.3% with GSK-LSD1 (Fig. 5f). These results suggest an important role for CACUL1 in mediating the reciprocal interplay between SIRT1 and LSD1.

### Gene expression profiling supports the role of CACUL1 in PPARγ-associated adipogenesis

**Fig. 6:**
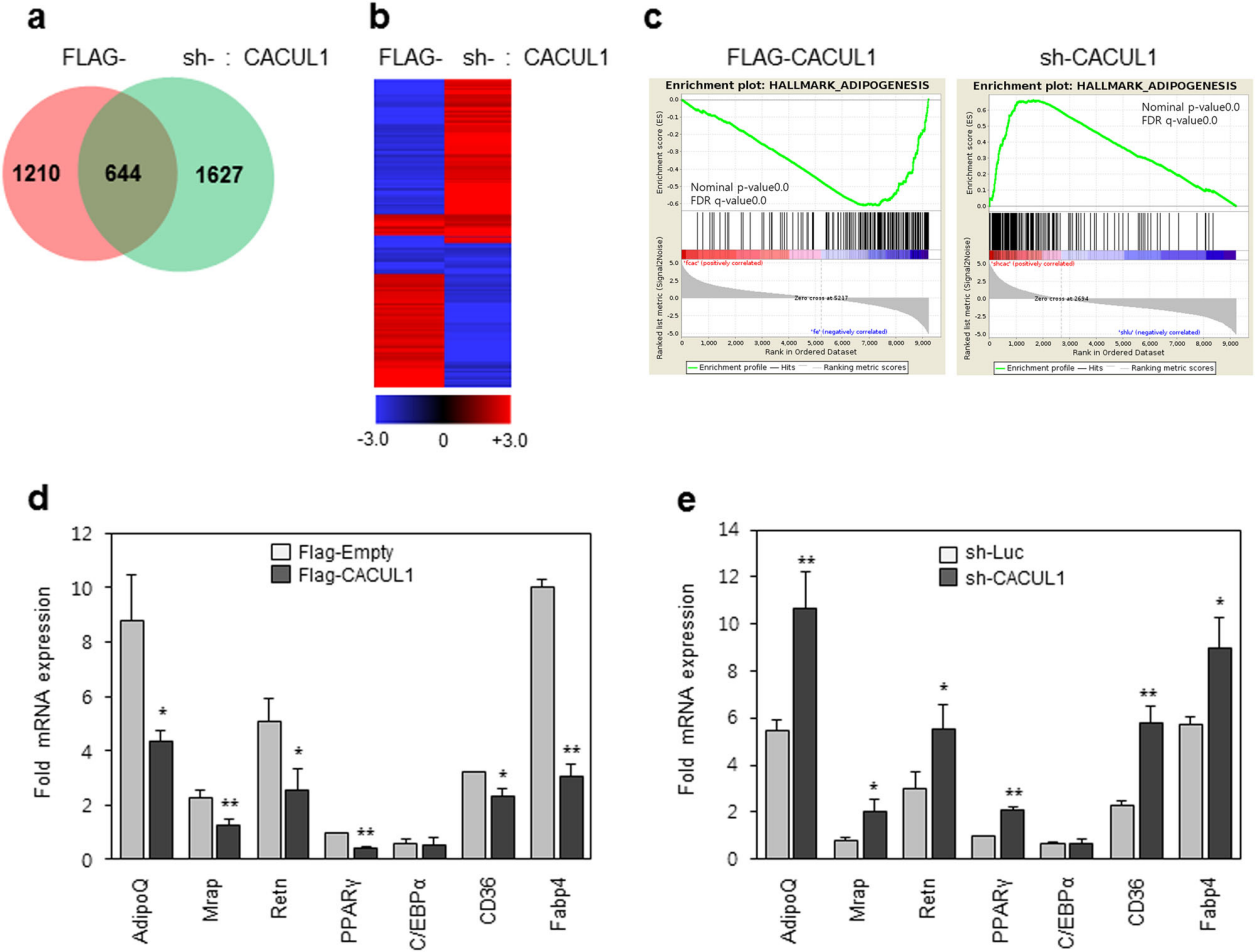
**Genome-wide analysis supports a critical role of CACUL1 in PPAR**γ **signaling** **a** Venn diagram of genes regulated by CACUL1. Stable 3T3-L1 cells with CACUL1 overexpression (FLAG-CACUL1) or knockdown (sh-CACUL1) were differentiated for 8 days and subjected to RNA-sequencing (RNA-seq). Genes with ≥2-fold expression changes were selected for each condition (FLAG-CACUL1, 1,854 genes; sh-CACUL1, 2,271 genes). **b** Clustering analysis. The 644 common genes were subjected to two-way hierarchical clustering. Data were satisfied with fc2 using Z-score for normalized values (log2 based). **c** Gene Set Enrichment Analysis (GSEA). One example associated with adipogenesis is shown. **d**, **e** Validation of RNA-seq data. Seven genes associated with adipogenesis and PPARγ signaling were selected and validated by RT-qPCR. Data indicate means±SD from three independent experiments. Fold mRNA expression was normalized to GAPDH expression (*P < 0.05, **P < 0.01)

To further define the function of CACUL1 in adipogenesis, RNA-seq was performed on differentiation day 8 using stable 3T3-L1 cells expressing FLAG-CACUL1 or sh-CACUL1. The number of genes with a greater than two-fold expression change under the conditions of CACUL1 overexpression and knockdown were 1854 and 2271, respectively (Fig. 6a). Subsequent gene ontology (GO) analysis showed that a high proportion of the regulated genes (>10%) were associated with cell differentiation, cell death, and cell proliferation under both conditions (Supplementary Figure S6A and B). Gene clustering analysis using 644 genes common to both conditions showed an opposite correlation between CACUL1 overexpression and knockdown (Fig. 6b and Supplementary Table S1). Additional GO analysis with these 644 common genes showed a strong correlation of CACUL1-regulated genes with adipogenesis, PPARγ signaling, and lipid metabolic processes (Supplementary Table S2). Of these common genes, 297 genes repressed by CACUL1 overexpression and activated by CACUL1 knockdown were responsible for the above GO data (Supplementary Table S3), supporting the role of CACUL1 in PPARγ repression. Consistently, gene set enrichment analysis (GSEA) indicated that adipogenesis-associated genes were downregulated under CACUL1 overexpression, and upregulated upon CACUL1 depletion (Fig. 6c). Further GSEA revealed that adipogenic targets of PPARγ were repressed in FLAG-CACUL1 stable cells but augmented in sh-CACUL1 cells. A similar finding was observed for adipogenic genes repressed by SIRT1 (Supplementary Figure S6C and D). Finally, we selected seven adipogenic genes known to be regulated by PPARγ and SIRT1, and measured their expression by RT-qPCR under the two CACUL1 expression conditions. All of the genes except *C/EBPα* were downregulated in CACUL1-overexpressed cells and upregulated in CACUL1-depleted cells (Figs. 6d, e). Overall, our genome-wide analysis suggests that CACUL1 is functionally correlated with PPARγ and SIRT1 signaling associated with adipogenesis.

### CACUL1 cooperates with SIRT1 to repress PPARγ function in human stem cells

**Fig. 7:**
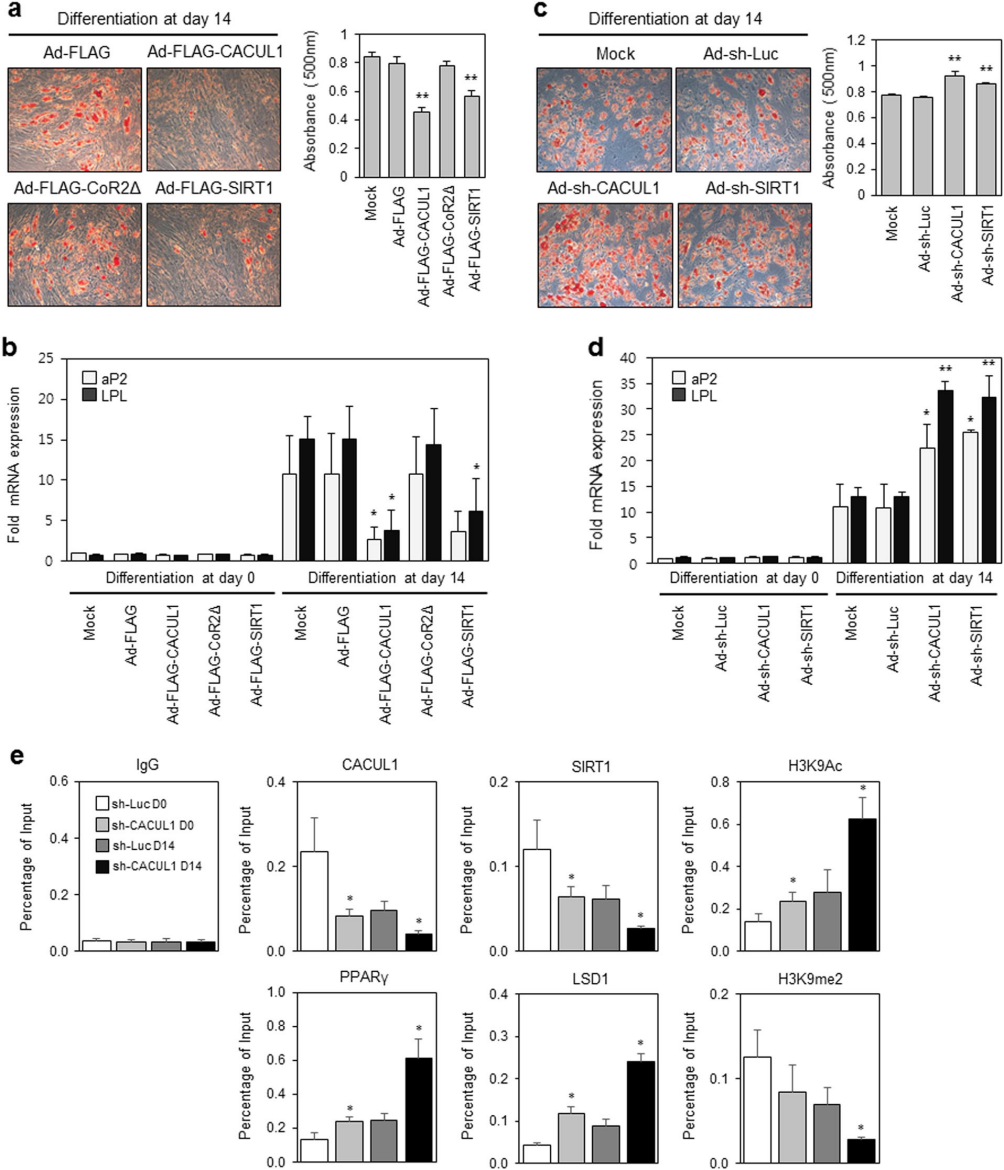
**CACUL1 cooperates with SIRT1 to repress PPAR**γ **function in human adipose-derived stem cells** (**ADSCs)** **a** Effects of CACUL1 or SIRT1 overexpression on differentiation of human ADSCs. ADSCs were infected with adenoviral expression vectors (FLAG-CACUL1, FLAG-CoR2Δ, or FLAG-SIRT1). After differentiation for 14 days, cells were subjected to Oil Red O staining and quantification of lipid content. Data indicate means±SD from three independent experiments (**P < 0.01). **b** Effect on the expression of PPARγ target genes. RT-qPCR was performed for *aP2* and *LPL* genes (*P < 0.05). **c** Effect of CACUL1 or SIRT1 depletion on differentiation. Data indicate means±SD from three independent experiments (**P < 0.01). **d** The effect of CACUL1 knockdown on the expression of PPARγ target genes (*P < 0.05, **P < 0.01). **e** The effect of CACUL1 depletion on H3K9 modifications during adipogenesis. After differentiation for 14 days, ADSCs infected with sh-CACUL1 were subjected to ChIP assays using a primer set specific for the proximal PPARγ-response site in the human *aP2* promoter and the indicated antibodies. Data indicate means±SD from three independent experiments (*P < 0.05)

Recent studies have reinforced the importance of SIRT1 for the self-renewal and differentiation of human mesenchymal stem cells^22^. Given that CACUL1 cooperates epigenetically with SIRT1 to inhibit PPARγ-mediated adipogenesis in mouse 3T3-L1 cells, we addressed whether CACUL1 modulates the differentiation of human stem cells. Adipogenesis was measured using an adenoviral expression system in ADSCs. Upon adipogenic stimuli, CACUL1 WT and SIRT1-expressing cells, but not CACUL1 CoR2∆ cells, exhibited a significant reduction in Oil Red O staining, which was quantified by measuring absorbance at 500 nm (Fig. 7a). The expression of CACUL1 WT, CoR2∆, and SIRT1 was measured using WB with an anti-FLAG antibody (Supplementary Figure S7A). The mRNA expression of PPARγ-responsive genes *aP2* and *LPL* was also strongly impaired by CACUL1 WT and SIRT1, but not by CoR2∆ (Fig. 7b).

To determine the effect of CACUL1 knockdown on ADSC adipogenesis, we infected ADSCs with sh-CACUL1 and sh-SIRT1 adenoviruses. Both CACUL1 and SIRT1 depletion clearly augmented adipogenic differentiation (Fig. 7c). The knockdown efficiency was also indicated by WB with an anti-CACUL1 or SIRT1 antibody (Supplementary Figure S7B). We also consistently observed increased expression of *aP2* and *LPL* upon CACUL1 or SIRT1 depletion (Fig. 7d). Finally, we measured the effects of CACUL1 depletion on the promoter occupancy of SIRT1 and LSD1 during adipogenesis in ADSCs using a proximal PPAR-responsive site within the human *aP2* promoter. Upon CACUL1 knockdown at differentiation day 14 (sh-CACUL1 D14), SIRT1 was less recruited than in control cells (sh-Luc D14), resulting in increased H3K9ac; whereas both PPARγ and LSD1 were more recruited than in controls, leading to reduced H3K9me2 (Fig. 7e). Binding site specificity was confirmed using a negative control (Supplementary Figure S7C). Consistent with the above 3T3-L1 data (Figs. 3–5), these results obtained using ADSCs confirmed that CACUL1 cooperates with SIRT1 (and negatively with LSD1) to modulate PPARγ repression, histone modification, and adipocyte differentiation.

## Discussion

Recently, epigenetics has emerged as a leading area of study for understanding the development of metabolic diseases, including obesity^23,24^. A variety of epigenetic changes accompany adipogenesis in preadipocytes (e.g., murine 3T3-L1 cells) or multipotent stem cells, finely controlling gene expression. Representative epigenetic marks include DNA methylation and histone modifications. Of these, complex networks of histone modifications (acetylation and methylation) are currently being investigated as the mechanism underlying adipocyte differentiation. Several histone-modifying enzymes reportedly regulate adipogenesis by producing either active histone codes, such as acetylated H3K9 and methylated H3K4, or repressive codes, such as methylated H3K9 and H3K27. Examples of these enzymes include: histone acetyltransferases (targeting the histone H3K9) promoting adipogenesis and histone deacetylases repressing adipogenesis^25^; H3K9 demethylase PHF2 promoting adipogenesis^26^; H3K9 methyltransferase G9a repressing adipogenesis^27^; H3K4 methyltransferase MLL4 promoting adipogenesis^28^; and H3K27 methyltransferase Ezh2 repressing *Wnt* genes to promote adipogenesis^29^. Although these enzymes are highly important for the fine control of adipogenesis, little is known about the cellular proteins regulating this enzymatic activity.

**Fig. 8:**
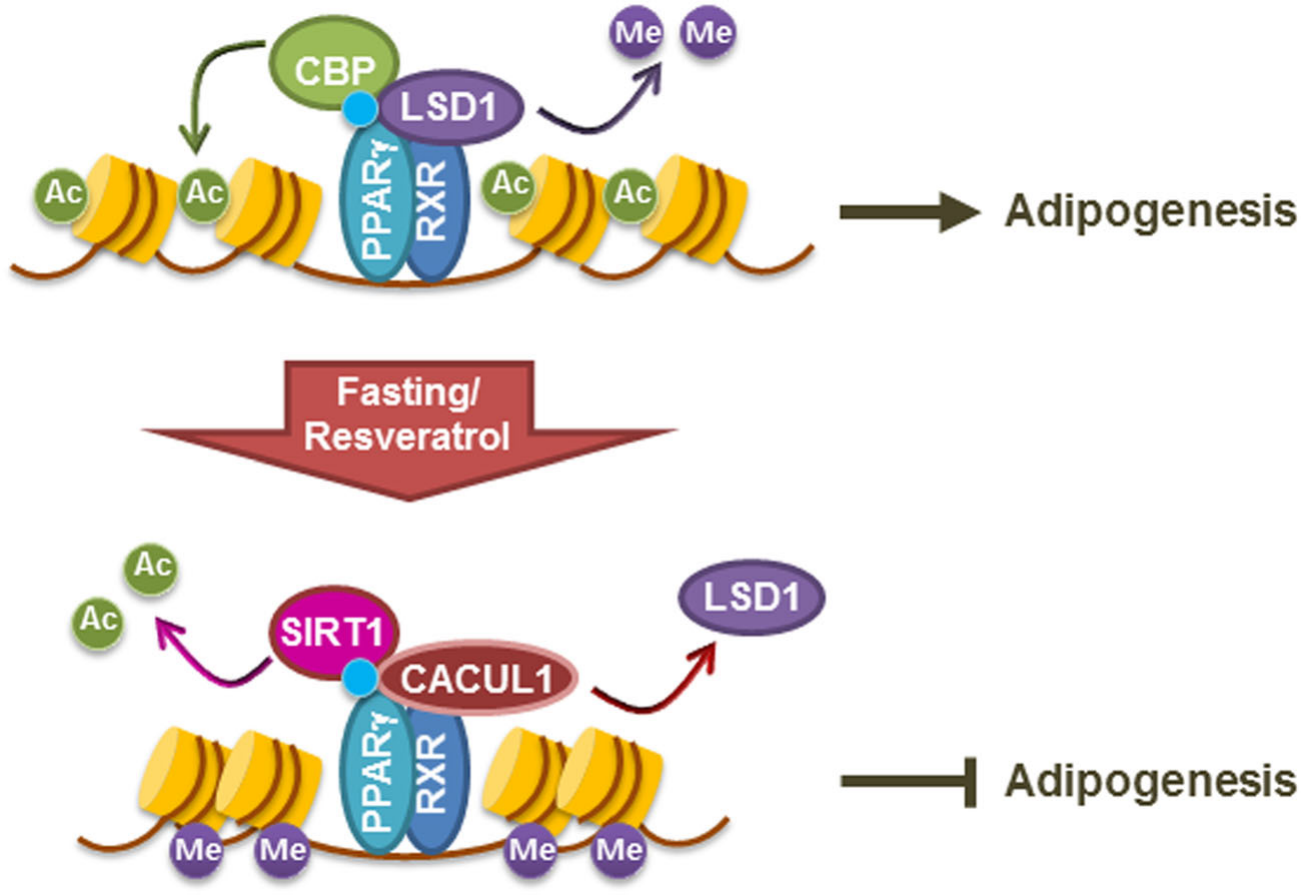
**Schematic model of CACUL1-mediated PPAR**γ **repression through opposite regulation of SIRT1 and LSD1** Under normal diet conditions, histone acetyltransferase CBP and demethylase LSD1 are recruited to PPARγ/RXRα-responsive genes, where they add an acetyl group to lysine 9 of histone H3 (H3K9) and remove methyl groups from di- or trimethylated H3K9, respectively, to activate the expression of adipogenesis-associated genes. Upon fasting (or glucose tolerance) or resveratrol treatment, SIRT1 and CACUL1 bind to PPARγ/RXRα-responsive genes, where LSD1 is dissociated by competing with CACUL1 and an acetyl group is cleaved from H3K9 by SIRT1, leading to inactivation of PPARγ target genes associated with adipocyte differentiation

In this study, we found that CACUL1 binds to PPARγ and represses its transcriptional activity via opposite regulation of SIRT1 (a deacetylase for histone H3K9) and LSD1 (a demethylase for H3K9) at the PPARγ target promoter, likely resulting in decreased adipogenic differentiation of murine 3T3-L1 preadipocytes and human ADSCs (Fig. 8). The repressive role of CACUL1 in adipogenesis-associated transcription was strongly supported by our RNA-seq-based expression analyses of whole genes (GO and GSEA). Previously, SIRT1 had been shown to mediate PPARγ repression by recruiting its coregulators, NCoR and SMRT, inhibiting adipogenesis^13^. Compared with this report, our data suggest diverse regulation of PPARγ activity and adipogenesis by adding CACUL1 and demethylase LSD1. To achieve PPARγ repression, CACUL1 cooperates with SIRT1 to reduce H3K9 acetylation, but interferes with LSD1 to enhance methylated H3K9 at the PPARγ target promoter (Fig. 5d, e). LSD1 is known as a histone demethylase specific for both mono- and dimethylated H3K4 and H3K9, thus playing a role in transcription repression (REST) and activation (ER, AR), respectively^30^. Considering the role of LSD1 in the demethylation of H3K9 during adipogenesis, we found opposite recruitment of LSD1 and dimethylated H3K9 depending on the level of CACUL1 occupancy, likely supported by the previous finding that LSD1 is dissociated from the promoter by incoming CACUL1, abolishing the LSD1-enhanced activation of ERα and AR^18,19^. Since the opposite roles of SIRT1 and LSD1 in regulating PPARγ activity and adipogenesis are quite obvious, it will be of interest to investigate whether a SIRT1 activator, such as resveratrol, can synergize with LSD1 inhibitors (GSK-LSD1) to suppress fat accumulation. Such a result would provide a baseline for the development of a combinatorial anti-obesity drug. Although data from murine 3T3-L1 preadipocytes and human ADSCs support each other, these molecular findings at the cellular level will be bolstered by generating CACUL1 knockout mice.

How is CACUL1 recruited to chromatin under fasting conditions or resveratrol treatment? SIRT1 is activated under these conditions. Activated SIRT1 forms a ternary complex with PPARγ and CACUL1, facilitating the recruitment of CACUL1 to PPARγ-responsive genes and thereby resulting in transcriptional repression. The physiological significance of CACUL1-mediated PPARγ repression under different dietary conditions is of potential interest. PPARγ expression induces adipogenic genes under energy-sufficient states such as high-fat dietary or adipogenic conditions. We observed that the expression of CACUL1 gradually decreased during the early stages of adipogenesis and increased during the later stages of adipogenesis (data not shown). Different expression levels of PPARγ and CACUL1 may reflect their roles in fine-tuning the adipogenic program during adipogenesis. Under fasting conditions, SIRT1 is activated to promote CACUL1 binding to PPARγ-responsive site without affecting CACUL1 expression (Fig. 5e), thus leading to cooperative repression of PPARγ activity. In contrast, under high fat conditions, PPARγ induces adipogenesis-associated genes by cooperating with coactivators including LSD-1 and CBP. Therefore, adipogenesis may be fine-tuned by dynamic regulation of PPARγ in response to different dietary conditions.

## Materials and methods

### Plasmids

All plasmid constructs were generated according to standard cloning methods and confirmed by sequencing as described previously^18,21^. The CACUL1 full-length and deletion mutants, SIRT1, LSD1, and PPARγ, were created by PCR amplification, and were inserted into a FLAG- or Myc-tagged pcDNA3 vector, GFP-fused pEGFP vector, or GST-fused pGEX4T-1 vector.

### Cell lines and culture

HEK293, HCT116, and 3T3-L1 cells were incubated in Dulbecco’s modified Eagle’s medium (DMEM) supplemented with 10% heat-inactivated fetal bovine serum (FBS) or bovine serum and an antibiotic−antimycotic mix (Life Technologies, Carlsbad, CA, USA). ADSCs (PT-5006; Lonza, Basel, Switzerland) were maintained in ADSC growth medium supplemented with FBS. All cells were cultured in an incubator at 5% CO_2_ and 37 °C. For stable cell lines, 3T3-L1 cells were transfected with FLAG, FLAG-CACUL1, FLAG-CoR2∆, sh-Luc, or sh-CACUL1 using the Lipofectamine^®^ Plus™ reagent (Life Technologies). The targeting sequence for CACUL1 knockdown (sh-CACUL1) was 5′-ATGTGTATGCCAGCAGCAC-3′. After 48 h, the cells were incubated with 0.8 mg/mL G418 (Life Technologies) for FLAG vectors or 0.1 mg/mL Hygromycin B (Life Technologies) for sh vectors. G418 or Hygromycin B-resistant colonies were selected for 2 weeks and stable gene expression was verified by WB using an anti-FLAG or anti-CACUL1 antibody.

### Recombinant adenoviruses

Recombinant adenoviruses were generated as previously described^31^. FLAG-CACUL1, FLAG-CoR2∆, FLAG-SIRT1, sh-luciferase, and sh-CACUL1 were constructed using the pAdEasy system. Adenoviruses were produced by transfection into 293A cells, and then cells were harvested. The final virus titer was measured by an Adeno-X™ Rapid Titer Kit (Clontech Laboratories, Mountain View, CA, USA).

### Yeast two-hybrid assays

Experimental procedures have been described previously^32^. The interaction was validated by yeast two-hybrid assays using LexA DNA binding domain (DBD)-fused SIRT1 and Gal4 activating domain (AD)-fused CACUL1. The level of interaction was determined using β-galactosidase (β-gal) and Bradford assays.

### Transient transfection and luciferase reporter assay

HEK293 or HCT116 cells were seeded in a six-well culture plate and transiently transfected with *aP2*-luciferase reporter and CMV β-gal expression plasmids. Polyethylenimine (Polysciences, Warrington, PA, USA) or Lipofectamine Plus reagent (Life Technologies) was used as transfection reagent, and cells were treated with 5 µm rosiglitazone for 24 h. Luciferase activity was observed using a luminometer (Promega, Madison, WI, USA) and normalized to β-gal activity.

### Immunoprecipitation and western blotting

Cell lysates were incubated overnight at 4 °C with the indicated antibodies diluted 1:200, and added to protein A/G agarose beads (Santa Cruz Biotechnology, Dallas, TX, USA). Immune complexes were released from the beads by boiling, and then analyzed by WB. Primary antibodies used in this study: α-FLAG (F1804; Sigma-Aldrich, St. Louis, MO, USA), α-Myc (05-724MG; Millipore, Billerica, MA, USA), α-GFP (sc-9996; Santa Cruz Biotechnology), α-SIRT1 (sc-15404; Santa Cruz Biotechnology), α-PPARγ (sc-7273; Santa Cruz Biotechnology), α-β actin (sc-47778; Santa Cruz Biotechnology), α-LSD1 (ab17721; Abcam, Cambridge, UK), α-H3K9ac (ab12179; Abcam), α-H3K9me2 (ab1220; Abcam), and α-CACUL1 (rabbit polyclonal antibody raised against amino acids 338–355; Peptron, Daejeon, South Korea). The WEST-ZOL^®^ system (iNtRON Biotechnology, Seongnam-Si, South Korea) was used for detecting protein bands.

### Glutathione S-transferase pull-down assay

GST pull-down assays were performed as previously described^19^. GST-fused CACUL1 was expressed in *Escherichia coli* and purified on glutathione Sepharose^®^ beads (GE Healthcare, Chicago, IL, USA). FLAG-SIRT1 or Myc-PPARγ protein was translated in vitro using the TNT^®^ Rabbit Reticulocyte System (Promega). Then, approximately equal amounts of GST or GST-CACUL1 were incubated with in vitro-translated FLAG-SIRT1 or Myc-PPARγ protein. Bound proteins were detected by WB using anti-FLAG or anti-Myc antibodies.

### Adipocyte differentiation and Oil Red O staining

Murine 3T3-L1 preadipocytes were grown to 100% confluence and then were cultured in adipogenesis initiation media containing 5 μg/mL insulin (I5500; Sigma), 1 µg/mL dexamethasone (D4902; Sigma), 0.5 mm IBMX (I7018; Sigma), with 5 μm rosiglitazone (R2408; Sigma) in 10% FBS DMEM. Starting at Day 2 (on Day 0), 10% FBS DMEM media containing 5 μg/mL insulin was added every 2 days. At the designated time, cells were fixed in 70% ethanol for 10 min and stained with the Oil Red O (Sigma) solution for 4 h at room temperature. Red-stained lipid droplets were observed by light microscope at ×100. Lipid content was quantified using a spectrophotometer at 500 nm. ADSCs were differentiated into adipocytes using the PGM™-2 Preadipocyte Growth Medium-2 BulletKit™ medium (Lonza) according to the manufacturer’s protocol.

### Reverse-transcription quantitative PCR

Total RNA from differentiated 3T3-L1 cells was extracted with TRIzol^®^ reagent (Life Technologies) according to the manufacturer’s protocol. Two micrograms of the total RNA was reverse-transcribed with random primers and M-MLV reverse transcriptase (Promega). RT-qPCR reactions were performed using the primer pairs shown in Supplementary Table S4. The mRNA expression was analyzed using SYBR^®^ Green Master Mix and the LightCycler^®^ system (all from Roche, Basel, Switzerland). All expression levels in each well were normalized using *GAPDH* as an internal standard. Fold expression was defined as the fold increase relative to controls.

### Chromatin immunoprecipitation analysis

ChIP assays were carried out as a reported previously^33^. Differentiated 3T3-L1 or ADSCs were cross-linked with paraformaldehyde. Chromatin-protein complexes were sheared and recovered by IP using the indicated antibodies. Cross-linking was then reversed according to instructions from Millipore. The DNA pellets were analyzed by RT-qPCR using primer pairs specific for PPAR-responsive regions (proximal and distal sites) and a non-PPAR target region (a negative control) within the *aP2* promoter (Supplementary Table S5). Percentage of enrichment compared to input was calculated and displayed.

### RNA-sequencing and bioinformatics analysis

Total RNA was extracted from controls (FLAG, sh-Luc), CACUL1-overexpressed (FLAG-CACUL1), and CACUL1-depleted (sh-CACUL1) 3T3-L1 cells after differentiation for 8 days. RNA samples were transferred to Macrogen (Seoul, Korea) for preparation of an mRNA-seq library, sequencing of the library, and isolation of differentially expressed genes. Results were filtered, and the cut-off was set at two-fold difference. Clustering analysis and heat map generation were performed with Multiple Experiment Viewer software (MeV 4.9.0). Genes exhibiting significant differences in expression level were classified into GO-based functional categories using the Gene Ontology Consortium (http://www.geneontology.org), the Kyoto Encyclopedia of Genes and Genomes (KEGG; http://www.genome.jp/kegg/), DAVID Bioinformatics Resources (http://david.abcc.ncifcrf.gov/), and MSigDB software v5.2 (http://software.broadinstitute.org/gsea/msigdb). The sequencing data used in this manuscript are deposited in the Gene Expression Omnibus (http://www.ncbi.nlm.nih.gov/geo/) under GEO accession number GSE105055.

### Gene set enrichment analysis

GSEA was performed with Java GSEA software v3.0 (http://www.broadinstitute.org/gsea). Normalized gene expression profiles were ranked with signal to noise metrics and enrichment scores were calculated with a random gene set permutation of 1000. Significance was set at nominal *P*-value (Nom *P*-value) < 0.05 and false discovery rat < 0.025.

### Statistical analysis

Statistical analyses are presented as means±standard deviation (SD) of at least three independent experiments. Comparisons between multiple groups were performed using paired *t*-tests. *P*-values < 0.05 were considered statistically significant.

## Electronic supplementary material

Supplementary information data

Supplementary Table S1

Supplementary Table S2

Supplementary Table S3
