# Supplementary material for: CACUL1 reciprocally regulates SIRT1 and LSD1 to repress PPARγ and inhibit adipogenesis
Source: Cell Death Dis. 2017 Dec 11;8(12):3201. doi: 10.1038/s41419-017-0070-z (PMC5870580; doi:10.1038/s41419-017-0070-z)
Supplement: Supplementary file 1 — Supplementary information data [file 41419_2017_70_MOESM1_ESM.docx]

**Supplemental information**

**CACUL1 reciprocally regulates SIRT1 and LSD1 to repress PPARγ and inhibit adipogenesis**

Min Jun Jang^1,3^, Ui-Hyun Park^2,3^, Jeong Woo Kim^1^, Hanbyeul Choi^1^, Soo-Jong Um^2^ and Eun-Joo Kim^1*^

*Correspondence: E-mail, [nbrejk@dankook.ac.kr](mailto:nbrejk@dankook.ac.kr)

Supplemental information includes Supplementary figures and legends (S1-S7), and Supplementary tables (S4 and S5).

**
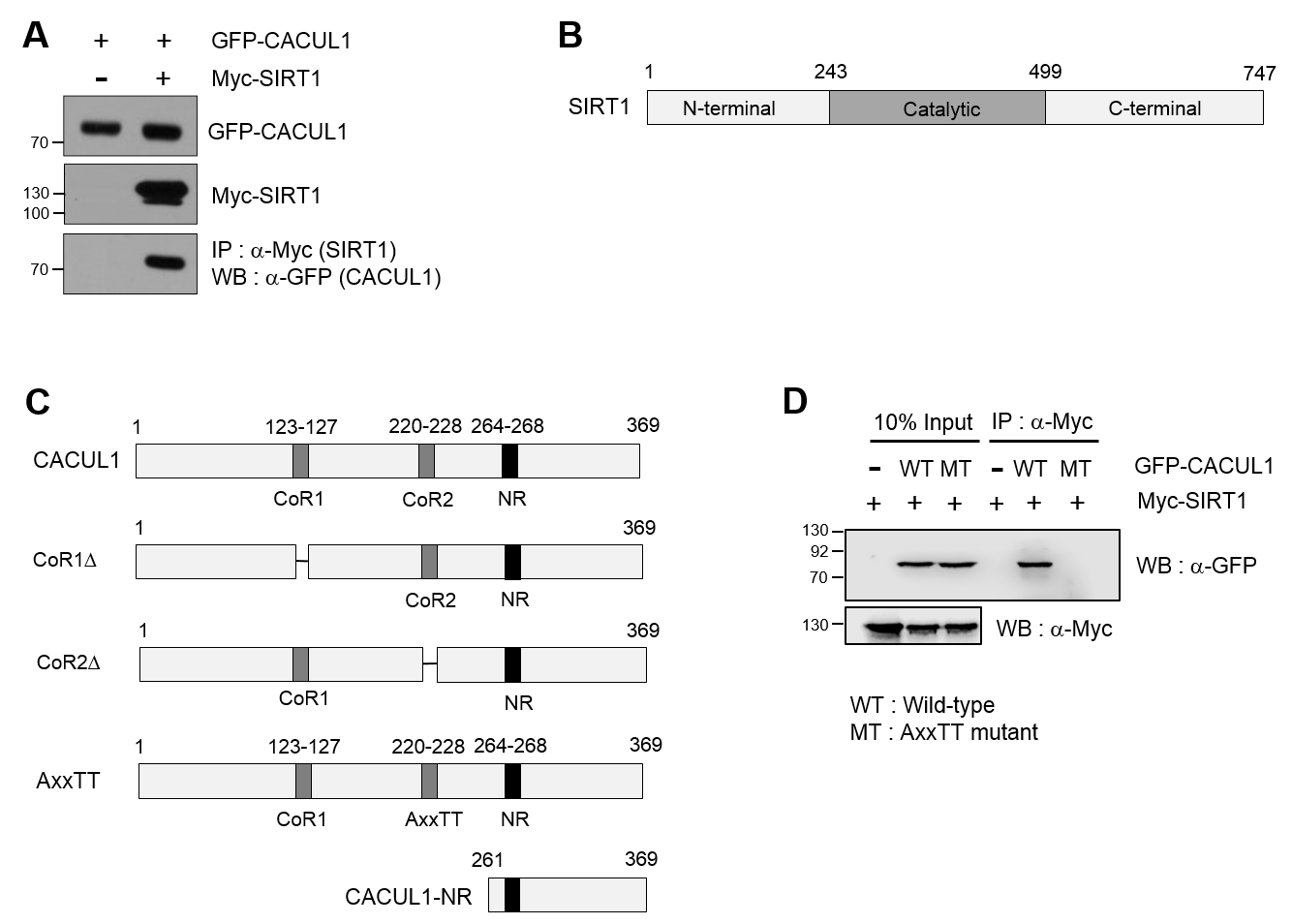
**

**Supplementary Figure** S**1. CACUL1 interacts with SIRT1.** (**A**) Co-IP analysis. HEK293 cells were co-transfected with GFP-CACUL1 and Myc-SIRT1 or the Myc vector. Immunoprecipitates recovered using an anti-Myc antibody were subjected to WB using an anti-GFP antibody. (**B**) Schematic representation of SIRT1 domains. (**C**) Schematic representation of CACUL1 motifs and mutants. CACUL1 lacking CoRNR box 1 (CoR1∆), CoRNR box 2 (CoR2∆), substitution mutant (AxxTT: amino acids LQSIV changed to AQSTT within the CoRNR box 2), and nuclear receptor box (NR) mutants are displayed. (**D**) The effect of the AxxTT mutant on SIRT1 binding. Co-IP and WB were performed as shown above except using HCT116 cells. WT and MT stand for wild-type and AxxTT mutant of CACUL1, respectively.

**
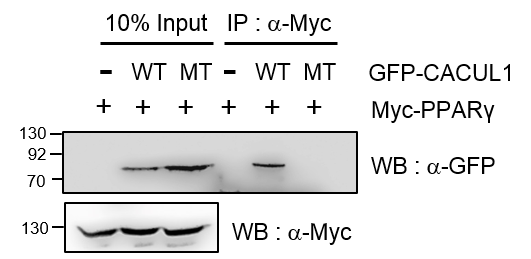
**

**Supplementary Figure** S**2.** The **CoRNR box 2 of CACUL1 is critical for PPARγ binding.** The effect of the AxxTT mutation on PPARγ binding was examined by co-IP and WB using anti-Myc (for PPARγ) and anti-GFP antibodies (for CACUL1 wild-type and AxxTT mutant) in HCT116 cells.

**
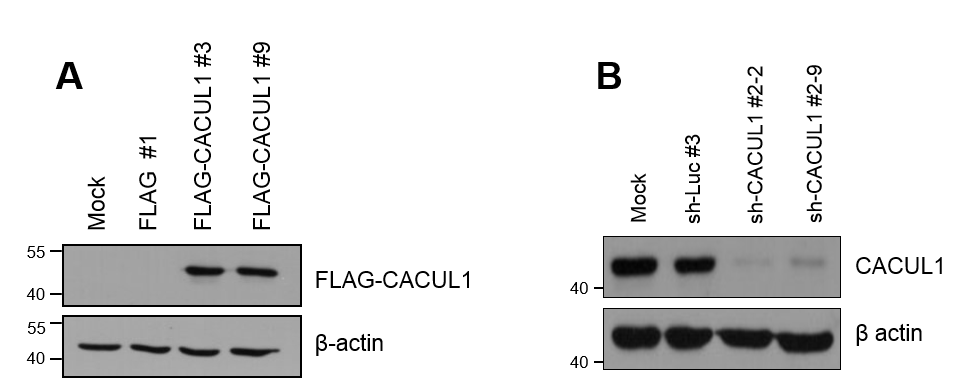
**

**Supplementary Figure** S**3. Expression of CACUL1 in stable 3T3-L1 cells.** (**A**) WB analysis of FLAG-CACUL1 stably expressed in 3T3-L1 cells. (**B**) WB analysis of CACUL1 after knockdown using sh-CACUL1.

**
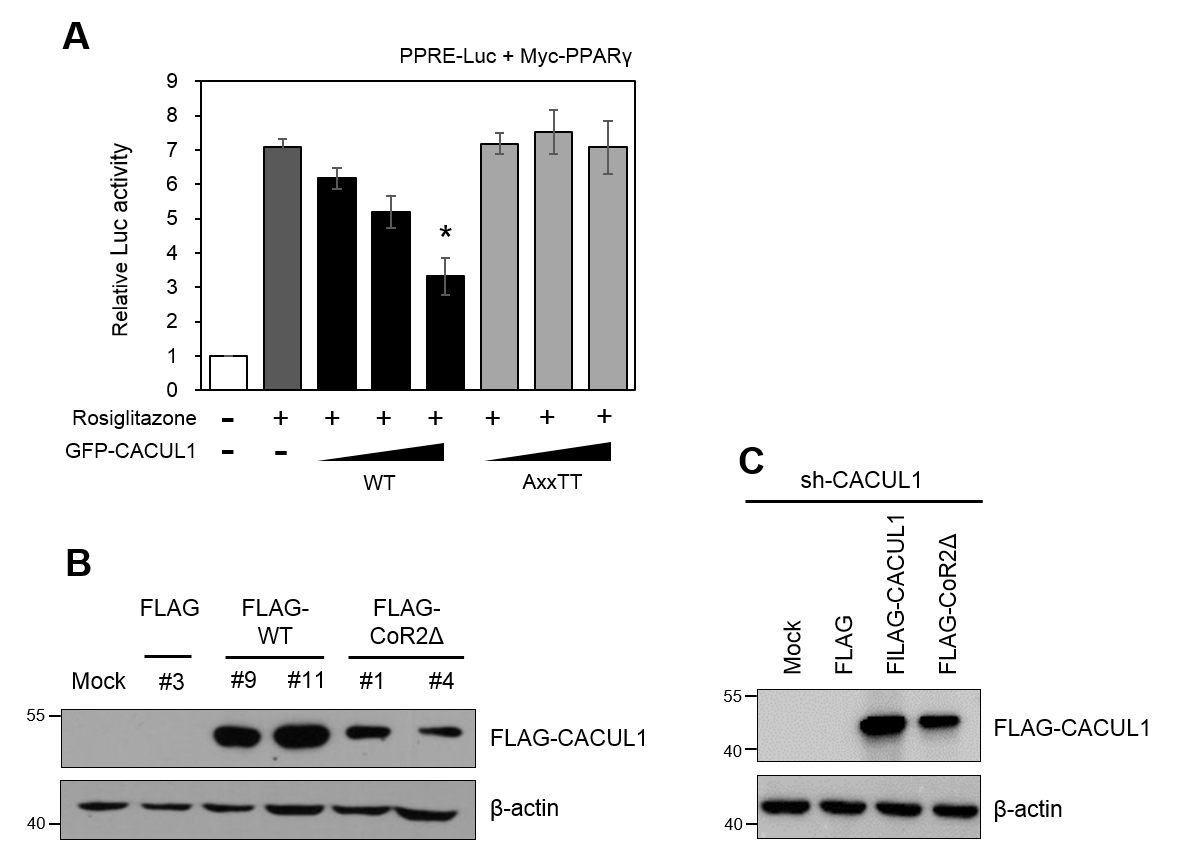
**

**Supplementary Figure** S**4. The CoRNR box 2 of CACUL1 is critical for PPARγ repression.** (**A**) Effect of the AxxTT mutant on the transcriptional activity of PPARγ. HCT116 cells were co-transfected with a PPRE-Luc reporter and increasing amounts of GFP-CACUL1 wild-type (WT) or GFP-CACUL1 mutant AxxTT together with Myc-PPARγ in the presence (or absence) of 5 μM rosiglitazone. Cell extracts were subjected to luciferase assays. Data are means ± SD from three independent experiments (*P < 0.05). (**B** and **C**) Expression of FLAG-CACUL1 WT and the CoR2∆ mutant in 3T3-L1 cells (**B**) and in CACUL1-depleted 3T3-L1 cells (**C**).

**
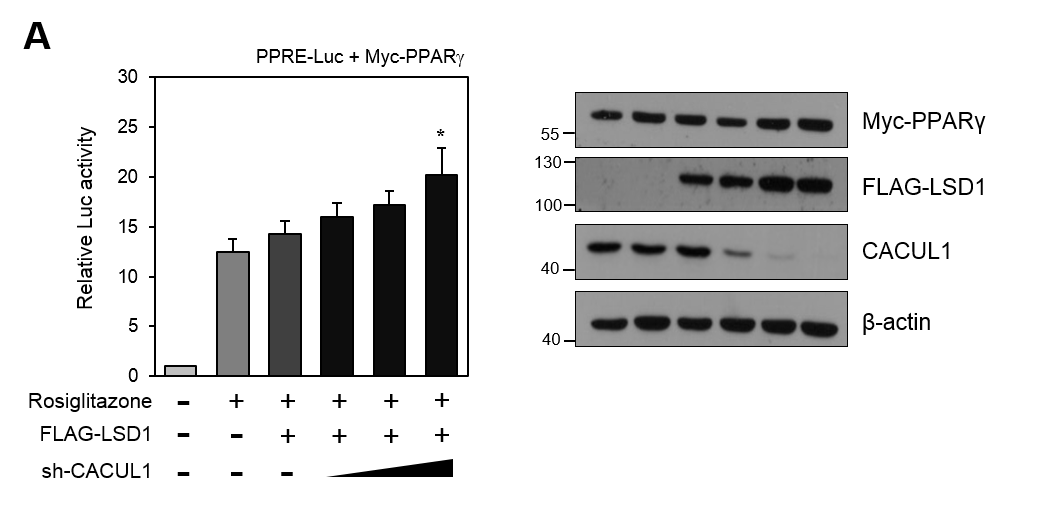

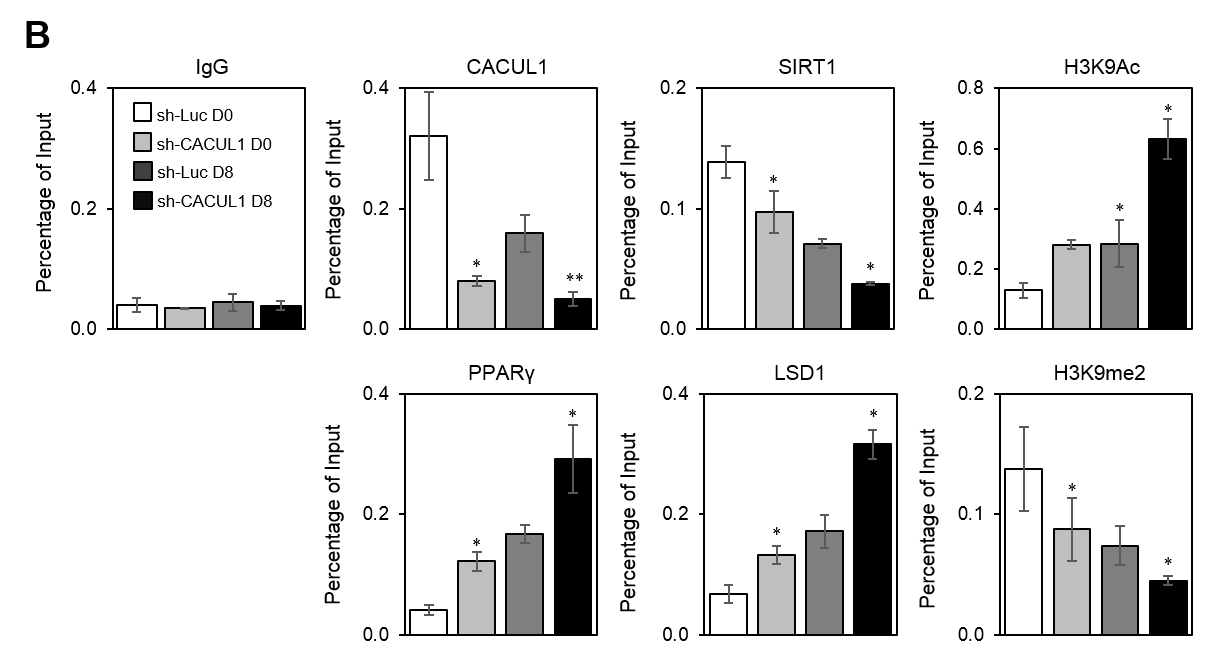
**

**
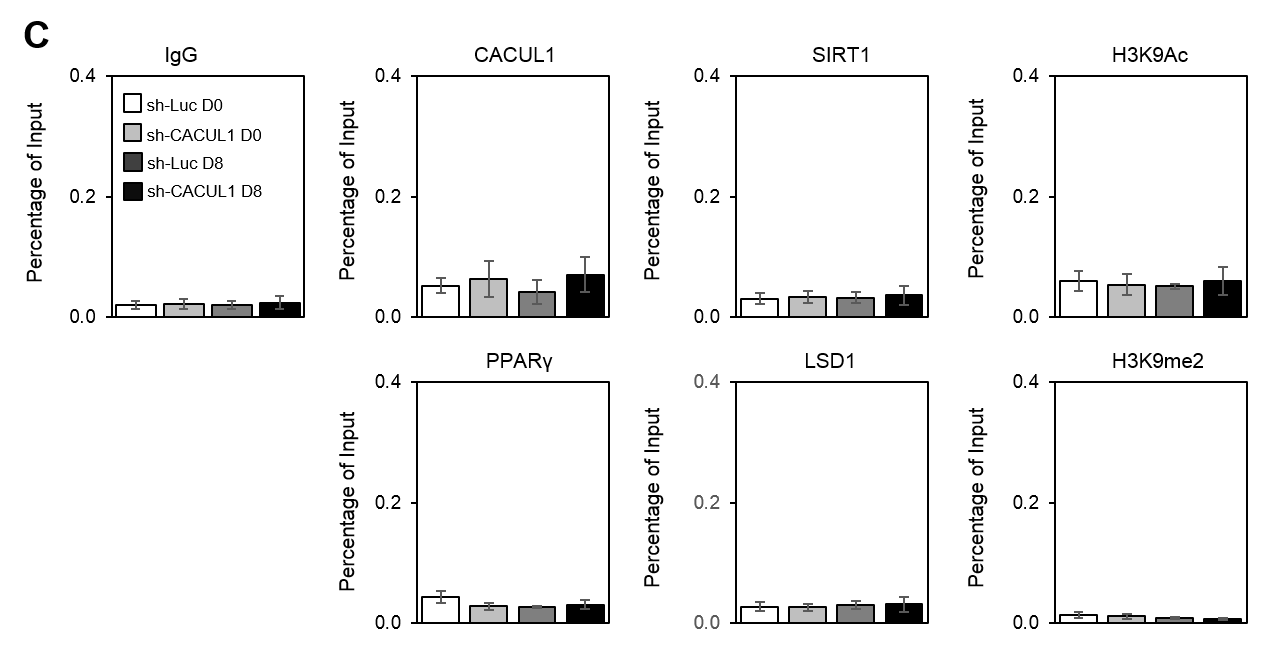
**

**Supplementary Figure** S**5. CACUL1 regulates SIRT1 and LSD1 opposingly at the** **PPARγ target.** (**A**) Effect of CACUL1 depletion on LSD1 activity. HEK293 cells were transfected with a PPRE-Luc reporter, Myc-PPARγ, FLAG-LSD1, and increasing amounts of sh-CACUL1 in the presence of 5 µM rosiglitazone. Cell extracts were subjected to luciferase assays. Protein expression was monitored by WB using the indicated antibodies. (**B** and **C**) ChIP analyses were conducted using the indicated antibodies and primer sets specific for the proximal PPARγ-response site (**B**) and a non-PPAR target site (**C**, negative control) within the murine *aP2* promoter in 3T3-L1 cells during adipogenesis.

**
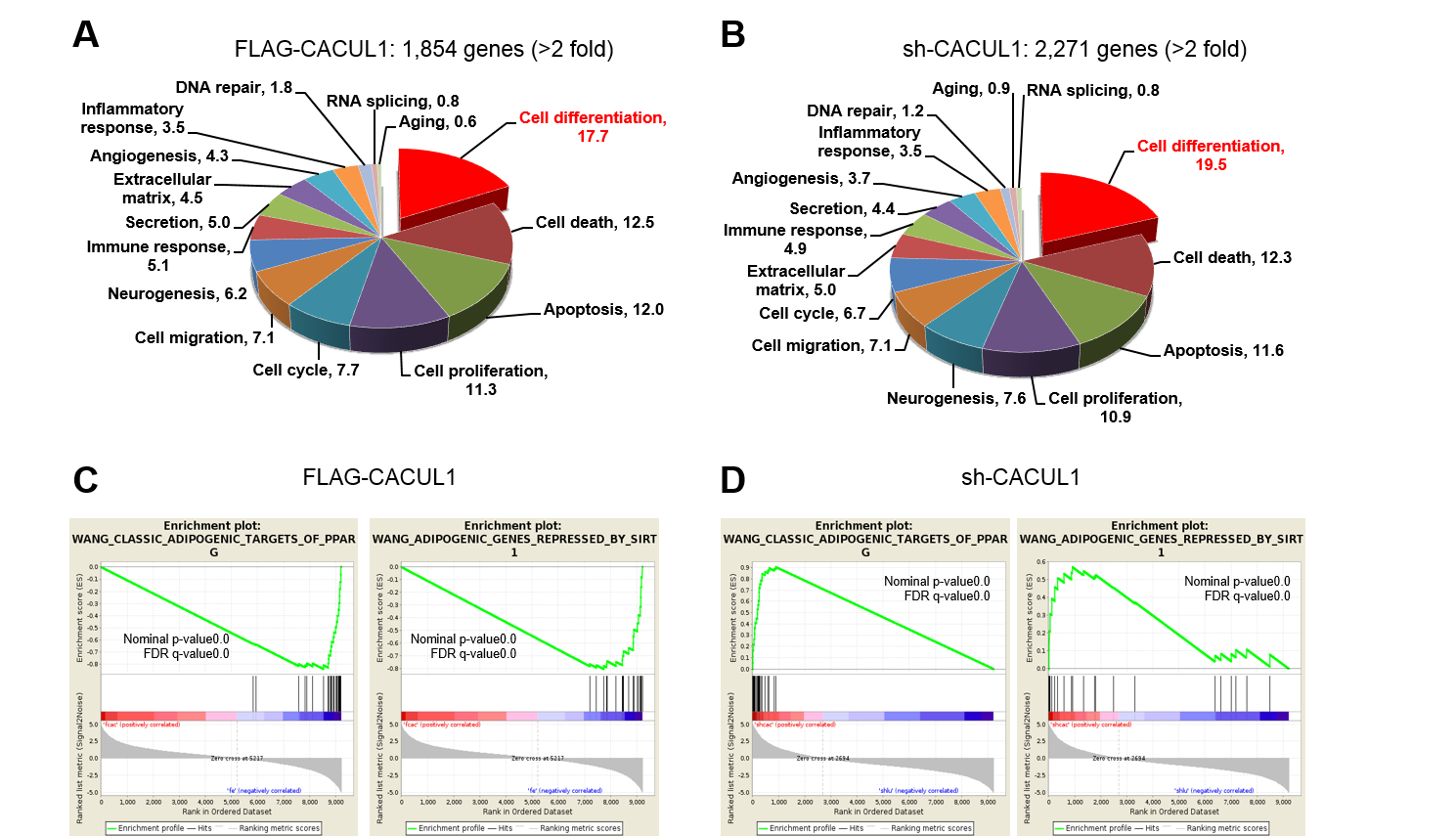
**

**Supplementary Figure** S**6. Gene ontology (GO) analysis and gene set enrichment analysis (GSEA) of genes regulated by CACUL1.** (**A** and **B**) Genes with ≥2-fold expression changes were selected under **CACUL1** overexpression (**A**) or knockdown (**B**) conditions and subjected to GO analysis. (**C** and **D**) GSEA was performed using whole genes altered by CACUL1 overexpression (**C**) or knockdown (**D**) to identify adipogenic targets of PPARγ and adipogenic genes repressed by SIRT1.

**
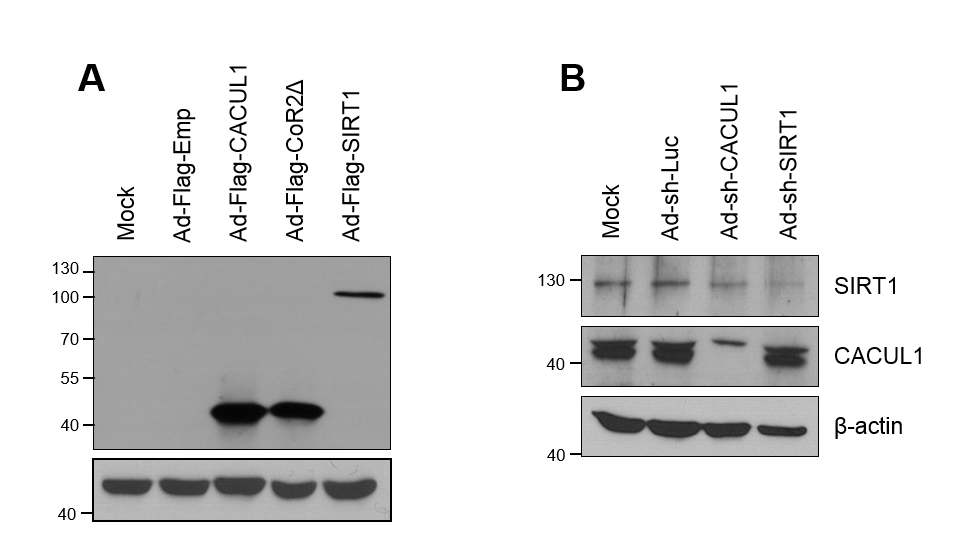
**

**
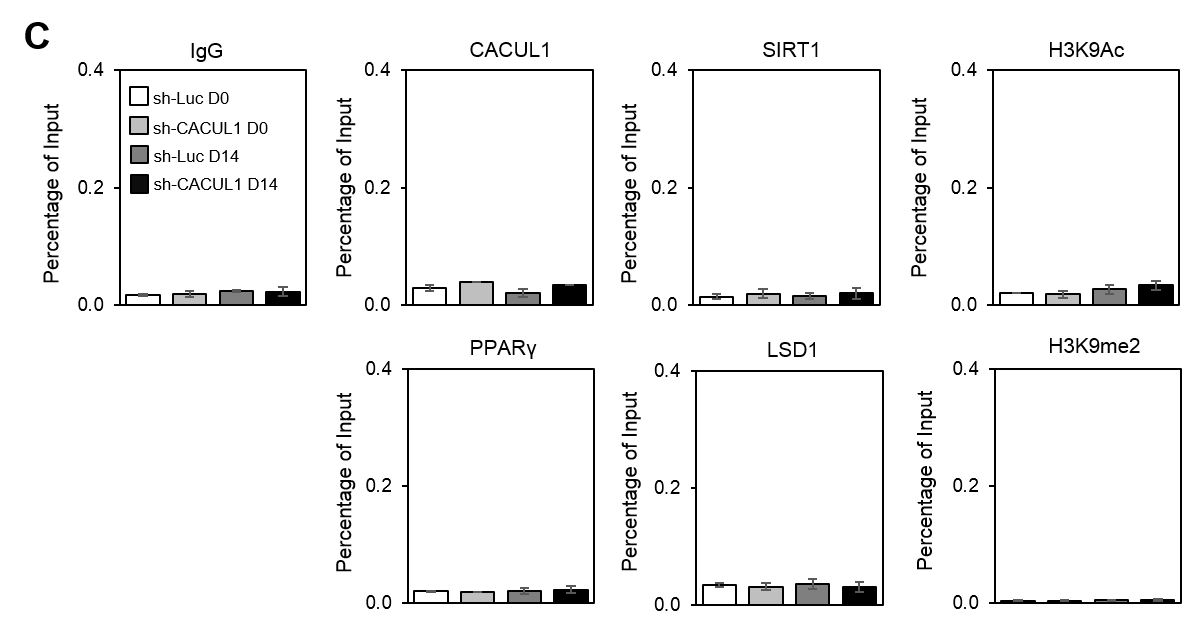
**

**Supplementary Figure** S**7.** **Analysis of human ADSCs.** (**A**) Expression of adenoviral expression vectors in ADSCs. (**B**) Expression of CACUL1 and SIRT1 after knockdown in ADSCs. (**C**) ChIP analyses using primer sets specific for a non-PPAR target site within the human *aP2* promoter and the indicated antibodies in human ADSCs during adipogenesis.

Supplementary Table 4. Primer sequences used for RT-qPCR

| Gene* | Forward primer (5’ to 3’) | Reverse primer (5’ to 3’) |
| --- | --- | --- |
| *maP2*  *(Fabp4)* | AAAGACAGCTCCTCCTCGAAGGTT | TGACCAAATCCCCATTTACGC |
| *mLpl* | ATCCATGGATGGACGGTAACG | ctggatcccaatacttcgacca |
| *mAdipoQ* | CGACACCAAAAGGGCTCAGG | CCAACCTGCACAAGTTCCCT |
| *mMrap* | CTGAAAGCCAACAAGCATTCCAT | AGAGGGAGGTTGAAGCTGTG |
| *mRetn* | TCAACTCCCTGTTTCCAAATGC | AGTTCTCAACTGACCGACATCA |
| *mPPARγ* | ATTGAGTGCCGAGTCTGTGG | GCCCAAACCTGATGGCATTG |
| *mC/EBPα* | TTCGGGTCGCTGGATCTCTA | TCAAGGAGAAACCACCACGG |
| *mCd36* | TGGGCAAGCAAGCTGTTCTA | CGTGGCCCGGTTCTACTAAT |
| *haP2* | AACCTTAGATGGGGGTGTCCTG | TCGTGGAAGTGACGCCTTTC |
| *hLPL* | CTGGACGGTAACAGGAATGTATGAG | CATCAGGAGAAAGACGACTCGG |
| *GAPDH* | CTGCACCACCAACTGCTTAGC | GGGCCATCCACAGTCTTCTGG |

*m, murine; h, human

Supplementary Table 5. Primer sequences used for ChIP analysis.

| Gene* | Forward primer (5’ to 3’) | Reverse primer (5’ to 3’) |
| --- | --- | --- |
| *maP2distal*  *(Fabp4)* | GAGCCATGCGGATTCTTG | CCAGGAGCGGCTTGATTGTTA |
| *maP2proximal*  *(Fabp4)* | AAATTCAGAAGAAAGTAAACACATTATT | ATGCCCTGACCATGTGA |
| *maP2negative*  *(Fabp4)* | CCAGTTTCTCAGCCTCCCTG | CAG ACT TTG GGG GCT GGT AG |
| *haP2proximal*  *(FABP4)* | TCCCAGTGTAGAGAGGGGAAA | GACAGGAGTGTCCCGAAGAG |
| *haP2negative*  *(FABP4)* | AAACACAGCCACCCTGACAT | GGACTCTCACTAACCCGTCG |

*m, murine; h, human
